# Cognitive testing of the Colon Cancer Screening Behaviours Survey with South Asian immigrants in Canada

**DOI:** 10.1186/s41687-017-0007-4

**Published:** 2017-10-19

**Authors:** Joanne Crawford, Farah Ahmad, Arlene S. Bierman, Dorcas Beaton

**Affiliations:** 1 Faculty of Applied Health Sciences, Department of Nursing, Brock University, 1812 Sir Isaac Brock Way, St. Catharines, ON L2S 3A1 Canada; 2 School of Health Policy and Management, Faculty of Health, York University, 4700 Keele St, Toronto, ON Canada; 3 Agency for Health Care Research and Quality, Washington, D.C., USA; 4 University of Toronto, 27 King’s College Circle, Toronto, ON Canada; 5 Musculoskeletal Health and Outcomes Research, Li Ka Shing Knowledge Institute, St. Michael’s Hospital, Toronto, ON Canada; 6 Measurement Stream, Institute for Work & Health, Toronto, ON Canada; 7 Department of Occupational Science and Occupational Therapy, Rehabilitation Sciences Institute, and the Institute of Health Policy, Management and Evaluation, University of Toronto, 27 King’s College Circle, Toronto, ON Canada

## Abstract

**Background:**

The purpose of this study was to cognitively test the Urdu and English language versions of a survey to assess colon cancer screening behaviours among South Asian immigrants in Canada.

**Methods:**

The Colon Cancer Screening Behaviours Survey was cross-culturally translated and adapted into the Urdu language followed by cognitive interviews using an evidence-informed cross-cultural cognitive interview framework. The cognitive interviews were conducted in English and Urdu in three rounds; a preliminary round, round one, and round two. Two bilingual cognitive interviewers administered interviews in person with South Asian immigrants in Hamilton, Ontario. Scripted verbal and emergent probe techniques were used concurrently with survey item administration.

**Results:**

A total of 30 South Asian immigrant participants, 12 English speaking and 18 Urdu speaking completed a cognitive interview. These groups were similar in age, gender, and years of residence in Canada. General design, culture, gender, and translation issues were identified. Revisions were made to improve the survey and the interview protocol was modified for future data collection.

**Conclusions:**

The cross-cultural cognitive interview framework led to a systematic and rigorous process of pre-testing and revising the Colon Cancer Screening Behaviours Survey, which may be used to gain insights on beliefs, benefits, facilitators and barriers to colon cancer screening among South Asian immigrants. The study methods and experience may also inform the cross-cultural translation and adaptation and cognitive testing of other survey tools.

**Electronic supplementary material:**

The online version of this article (doi:10.1186/s41687-017-0007-4) contains supplementary material, which is available to authorized users.

## Background

In order to understand colorectal cancer (CRC) and screening behaviours among South Asian (SA) populations, an understanding about socio-cultural context, facilitators and barriers to participation from their perspective is required. Knowledge gained may then be used to inform selection of relevant measures to assess the factors that influence CRC screening.

Prior studies were conducted by our team to inform the development of a survey targeted for use with SA immigrant populations to assess beliefs, attitudes, facilitators and barriers to CRC screening [1, 2]. A scoping study enhanced understanding of factors that influenced cancer screening among SA immigrants residing in the United Kingdom (UK), the United States of America (USA), and Canada [1]. This initial work provided knowledge on socio-cultural context including values of family, holistic views of health care, beliefs related to risk perception, low knowledge, and barriers to screening. However, the scoping study was limited in terms of understanding beliefs, barriers, and gender-related factors that influence CRC screening among both men and women [1]. This gap was addressed by conducting a focus group study to elicit the perspectives of SA immigrants to gain a more focused understanding of CRC and screening perceptions within the Canadian context [2]. The findings informed on socio-cultural beliefs and attitudes related to CRC and screening, as well as provided an enhanced understanding of sources of knowledge and awareness from social networks. Additionally, the factors that supported access to CRC screening uptake were uncovered along with key strategies to promote CRC screening in SA communities. In summary, both studies were essential to inform the development of a survey to assess CRC screening behaviours among SA immigrants.

Survey development followed a rigorous process [3] beginning with the identification and charting of concepts extracted from prior studies [1, 2]. Key concepts were then defined using behavioural concepts from the Health Belief Model [4] and the Theory of Planned Behaviour [5]. A literature review of articles reporting on pre-existing surveys containing measures that aligned with key concepts and conceptual definitions were selected and critically appraised for conceptual congruence using the Evaluating the Measurement of Patient-Reported Outcomes tool [6]. Consultation with public health and measurement experts enabled the selection of candidate measures and decision-making around additions and modifications required within the survey. This initial phase of survey development was important for survey construction as it followed a rigorous process.

The Colon Cancer Screening Behaviours Survey consists of 84 items that assess: (1) CRC screening practices and behavioural outcomes; (2) six scales that measure *perceived susceptibility*, *perceived severity*, *perceived benefits*, *perceived barriers*, *self-efficacy*, and *subjective norm* from existing instruments; and, (3) relevant socio-demographics for SA populations [3]. See Table 1 for the Summary of Measures in the survey that includes details on domains and items, pre-existing measures, and additions from the literature. A total of five items were researcher developed. The purpose of the survey is to describe or predict colon cancer screening beliefs, attitudes, facilitators, and barriers that influence intention and uptake among SA immigrants in Ontario, Canada. As developed, the survey was intended for in-person or telephone interviewer-led administration; although, it may be used for self-administration in educated populations. The survey was translated into Urdu to be accessible to non-English language speaking individuals. At this stage of pre-testing, we did not feel it was necessary to obtain permission from the authors of pre-existing measures included in the survey as we did not know if further modifications would be required until after the study was completed. The intent was to contact authors after this study to seek permission upon pilot testing of the survey.

**Table 1:** Summary of Measures in the Survey

Domains	Pre-existing measures	Additions
Colon cancer screening practices – Total: 9 Items		
Heard, had, and intention for colon cancer screening	Vernon et al. [37]	3 items added
		• 2 items drawn from prior literature [43] • 1 item - researcher developed
Colon cancer screening beliefs and attitudes - Total: 60 Items		
*Perceived susceptibility* & *Perceived severity*	*Perceived susceptibility* and *perceived severity*	*Perceived susceptibility*
	Ozsoy et al. [31] used Jacobs [44] measures for colorectal cancer screening that were initially drawn from Champion’s [45] breast cancer screening measures	• 1 item was added [46]
		*Perceived severity*
		• 2 items added [47, 48]
*Perceived benefits* & *Perceived barriers*	*Perceived benefits* and *perceived barriers*	*Perceived benefits*
	Rawl et al. [49] colorectal cancer screening measures were drawn from Champion’s [50] breast cancer screening measures	• 2 items: 1 item for the home stool test and colonoscopy [51]
		*Perceived barriers*
		• 3 items were added [34, 52]
*Perceived self-efficacy* & *Subjective norm*	*Perceived self-efficacy* and *subjective norm*	*Perceived self-efficacy*
	Flight et al. [53] used measures from Tiro et al. [54] and Vernon et al. [55]	• 1 item was added [56]
		*Subjective norm*
		• 1 item - researcher developed [1]
Socio-demographics – Total 15 items		
Socio-contextual items relevant to SA immigrants	Items drawn from scoping and focus group studies [1, 2]	4 items - researcher developed [1]

Current standards of linguistic translation [7] of a survey alone may be insufficient to achieve relevance to target respondents [8, 9]. For new immigrants, attention must be paid to achieving conceptual equivalence while being sensitive to socio-cultural context. Although this may be addressed in good cross-cultural translation and adaptation, it should be verified using cognitive interviews [10]. Pre-testing with cognitive interviews may uncover issues with comprehension of questions and responses, design layout, or translation [10]. This was the case for Pasick et al. [11] in their study assessing breast and cervical cancer screening among diverse ethnic populations in the USA, where conceptual, translation and contextual issues arose.

The purpose of this study was to conduct cognitive interviews of the English and Urdu versions of the Colon Cancer Behaviours Screening Survey with SA immigrants. Our objective was to assess the English and Urdu language versions of the survey for comprehension through probing, to interpret qualitative data and key themes, and to identify problems with questions and responses while comparing issues that emerged among each group, and to make revisions.

## Methods

### Cross-cultural translation and adaptation

**Fig. 1:**
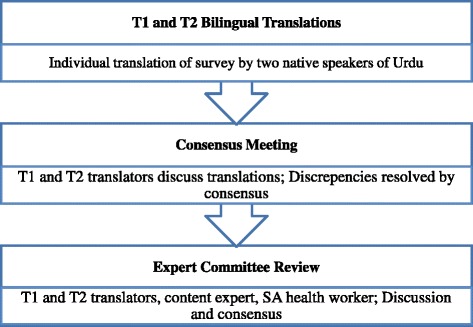
Cross cultural Translation and Adaptation. Beaton et al. [8]; Epstein et al. [9]

The Colon Cancer Screening Behaviour Survey was cross-culturally translated and adapted into the Urdu language using current best practices by two bilingual SA immigrants, and involved: (1) two independent forward translations; (2) a meeting to discuss the synthesis report of the two forward translations; and (3) an expert committee review meeting ([8, 9], See Fig. 1). The decision to forgo back-translation, a common second step in cross-cultural translation and adaptation was made because of the literature reporting that expert committee review produces more accurate cross-cultural translation and adaptation (i.e. face validity and conceptual relevance) when compared to back-translation [9] Epstein et al. [9] described that back translators could return the survey to the original wording skipping over incorrect terms in the forward translation, and thus provide unwarranted confidence in the translated version. The committee review provided an opportunity to involve two other individuals with expertise working with the SA community, and who had greater understanding of medical terms and how they may be interpreted or understood by the target population.

The first step required two translations (T1 and T2) from two individual translators. These individuals were bilingual, emigrated from the Indian sub-continent, and were native speakers of Urdu; one had an undergraduate degree and lay knowledge of the topic, and another had a health science degree and background. Documentation was provided on each translation. In step two, a synthesis of the two independent translations (T1 and T2) was created and documented. Discrepancies were resolved by consensus through the process of returning to the synthesized documentation report, and discussing the issue among the two translators. The source and target versions of the survey were examined using the following criteria: (a) semantic equivalence, the meaning ascribed to words used by SA populations; (b) idiomatic equivalence, the idioms or colloquialisms used by the SA population; (c) experiential equivalence, the context of the activity, CRC screening; and (d) conceptual equivalence, the meaning of concepts examined and defined within the culture [8, 9].

The next step involved an expert committee review of the combined translations (T1 and T2) synthesis report of the survey including written documentation that outlined decision-making processes that took place during translation [9]. To avert problems related to accurate linguistic and cultural translations of key medical terms during expert committee review, a bilingual clinical expert and a content expert in cancer screening and public health informed this final step [12]. Therefore, the expert committee was comprised of the two individual translators, a bilingual SA clinical expert, and a content expert. The expert committee played a vital role in the final step of cross-cultural translation and adaptation before pre-testing the survey. This was accomplished through discussion of each individual’s interpretation, and reaching a consensus on any issues or discrepancies. To log results of cross-cultural translations, three standardized forms were used, one for each step [13].

### Cognitive interviews

The survey was then pre-tested using cognitive interviews. Cognitive interviewing has its foundations in social and cognitive psychology and focuses on an individual’s cognitive processing of questions and responses [14]. Testing the function of a survey using cognitive interview methods is an important step in evaluating a newly developed survey questionnaire [15]. The overall aims are to reduce systematic error, and improve the functioning of the measures in the target culture [16]. The Cognitive Interviewing Reporting Framework provides guidelines for the reporting of studies involving cognitive testing, and is used in this paper [17].

#### Cognitive and cross-cultural interview framework

Cognitive interviewing was conducted using a question-and-answer process [14]. The intent was to uncover problems by probing the participant about each question to determine whether a specific question did or did not successfully match the intended concept of interest. The techniques detect issues of comprehension, processing or communication, and serves to complement subsequent field testing [14]. In our study, the development of scripted *verbal probes* was guided by Tourangeau’s [18] theoretical framework. This framework utilizes a question-and-answer model with four processing actions to respond to a question: comprehension, retrieval, judgement, and response. The objective of cognitive interviews in this study was to provide opportunities to elicit evidences of these cognitive processes and uncover any conceptual issues.

The cross-cultural relevance was enhanced by drawing from Willis’ cross-cultural cognitive interviewing, a variant of standard cognitive interviewing [10]. A unique feature of the cross-cultural cognitive interviewing is that it assesses cross-cultural equivalence between the source language and target language survey, and aims to determine if the variation of interpretation of items between both is acceptable given the measurement goals.

#### Specific techniques

Scripted and emergent *verbal probes* were used in this study as it placed less responsibility on the participant [16, 19, 20], and provided a structured method for use by interviewers with limited cognitive interviewing skills. *Verbal probing* is used to probe a specific question, a term, or the path that led to the response [16, 21]. Standardized construction of scripted *verbal probes* was guided by investigator consultations with a public health advisory group, and other measurement experts [16]. In total, there were 12 scripted *verbal probes* (Additional file 1: Appendix A).

Using a standardized process, the interviewer was responsible for following the flow of questions, responses, and scripted *verbal probes* using a protocol. Emergent *verbal probes*, another form of *verbal probing* were also used to uncover unanticipated problems. The cognitive interviewer was attuned and observed the participants’ hesitation or confusion with a question or response during the interview and as a result formulated *verbal probes* needed to elicit further information [16]. Emergent *verbal probing* has been found to be effective as a testing method among a variety of cultures and language groups [10]. The interviewers followed the cognitive interview protocol with concurrent probing where each item of concern was administered and followed by scripted *verbal probes* [16].

The addition of cross-cultural cognitive interviewing allowed us to pre-test the Colon Cancer Screening Behaviour Survey among SA immigrants to: (1) assess previously developed and tested items; (2) assess if it was understood in the same way through the administration of the Urdu and the English language version of the survey; and (3) identify problems.

#### Cognitive interview study design

The Colon Cancer Screening Behaviours Survey was cognitively tested through interviewer-led in person administration using pen and paper in Hamilton, Ontario. The participant eligibility criteria included: 50 to 74 years of age, average risk for CRC (no personal or family history of CRC, no inflammatory bowel diseases, no symptoms or bowel problems [22]; country of birth in the Indian sub-continent or SA *diaspora*, permanent residency; and, Urdu and English language speaking. Interviews were held separately for English and Urdu language speaking participants. We aimed to recruit a purposive sample of 30 participants. Given the two participant groups for interviews differed by language only, this sample size was determined to be reasonable to meet study objectives [16, 21, 23], and to reach saturation for qualitative assessment [24]. Research Ethics Board (REB) approval was received from two universities that the primary author was affiliated with during the time of the study, the University of Toronto Research Ethics Board (# 27857), and Brock University Research Ethics Board (#12–036). The other authors were also affiliated with the University of Toronto during the time of the study.

#### Procedures

**Fig. 2:**
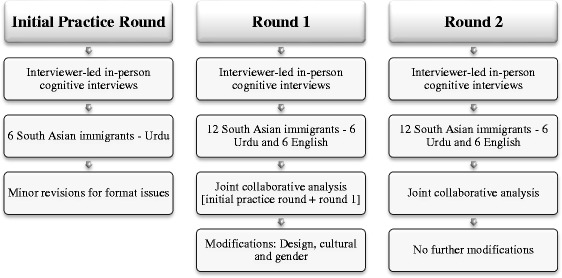
Cognitive Interview Rounds

Pre-testing using cognitive interviews were conducted in three rounds: an initial practice round, round one and round two, similar to the process reported by Levin and colleagues ([20], See Fig. 2). The initial practice round used the Urdu survey and was meant to provide both a source of training for the cognitive interviewer and the opportunity to modify the cognitive interview protocol if needed. With minor format and wording edits made to the cognitive interview protocol after the practice round (Urdu only), we proceeded to round one using the Urdu and English language surveys. Round one consisted of 12 cognitive interviews with SA immigrant participants: six with the Urdu language survey and six with the English language survey. After round one *joint collaborative analysis* and modification to the cognitive interview protocol, round two cognitive interviews were conducted with 12 SA immigrant participants; six with the Urdu language survey and six with the English language survey (See Fig. 2). Revisions made after round one led to round two in both languages. After round two, similar beliefs and ideas were repeated and no major problems emerged, and thus we decided no further rounds were required. We stopped at round two because we believed that the cognitive testing was sufficient to improve the survey prior to field testing.

Participants were recruited from community contacts and/or snowball sampling in select Hamilton, Ontario communities where SA immigrants lived, worked, or congregated. The cognitive interviewers were also trained to use purposive sampling via word-of-mouth to recruit participants [25]. During participant recruitment, attempts were made to recruit participants with varying years of residence in Canada [26]. One cognitive interviewer had greater access to diverse SA populations who had limited English language proficiency; therefore, she recruited Urdu language speaking participants. The other cognitive interviewer recruited English language speaking participants as she had greater access to this population.

Prior to each interview, written informed consent was obtained from SA immigrant participants. The interviews were conducted in the evening or weekend days at participants’ homes. Audio or video-taping of interviews was not feasible or culturally appropriate. A $20.00 honorarium was given to each participant.

#### Cognitive interviewers and training

Two bilingual research assistants conducted cross-cultural cognitive interviews. Cognitive interviewer A, a lay community member with prior experience working in the SA immigrant community resided in Canada for 15 years. Cognitive interviewer B, a gynaecologist in her native country (Pakistan) and in training for medical credentialing in Ontario, resided in Canada for approximately 2 years. Both cognitive interviewers had no prior cognitive interview experience or training.

Initial training of cognitive interviewers took place at a community center for 3 h with an additional 1 h provided throughout the testing rounds. Training material covered how to administer survey questions exactly as worded in English, or as they were translated in Urdu. Content in training encompassed: (a) the use of scripted *verbal probes*; (b) data collection procedures; (c) the observation and detection of problems or issues through non-verbal behaviours and use of emergent *verbal probes*; (d) the interview process and a mock demonstration; and (e) *joint collaborative analysis* using Willis’ [16] process. Cognitive interviewers’ were instructed to be clear, unbiased or leading when using scripted and emergent *verbal probes* [16].

The cognitive interviewers were trained to adhere to the cognitive interview protocol process. The cognitive interviewers were supported in the field by the lead researcher. They probed participants concurrently making detailed annotations of responses. The cognitive interviewer who conducted interviews in the Urdu language completed two surveys, one in Urdu, and one in English for all rounds of testing for 18 surveys. She conducted the interview in Urdu while simultaneously taking notes in Urdu, and then documenting responses on the English language survey protocol. This process was important to be able to return to notes during *joint collaborative analysis* for any clarification if required. Any critical issues from the target Urdu language were documented to assess language and translation, or other related issues. The cognitive interviewer who conducted interviews in the English language made notations on one document for 12 surveys.

#### Data Collection & Analysis

Analysis of data from the practice round, round one and round two was conducted using a *joint collaborative analysis* process involving data reduction and interpretation [27]. This process involved each cognitive interviewer reviewing results after the interview, and then meeting with the lead researcher to discuss individual results. This allowed a joint process of sharing information and interpretation so that all were involved in determining question functioning at the individual level. Willis’ [16] process for data analysis was used to undertake *joint collaborative analysis*.

The first step involved the use of the cognitive interview protocol as a recording tool to write participant responses and comments. This data formed the “case history” and the lead researcher was responsible for collating all summary data into an electronic copy using a participation identification code under each item [16]. The next step entailed the aggregation of results by each interviewer from all individual interviews by items to assess if there were common themes. This was facilitated as a *joint collaborative analysis* meeting with both cognitive interviewers and the lead researcher. The meeting enabled all members to be involved in the discussion of results at three separate meetings for the practice round, round one, and round two. Results of interview data for the round of testing were examined together to enable discussion across all interviews; first overall, and then item-by-item separately for the Urdu and English language interviews. The goal was to observe patterns or recurrent themes in the identification of problems, similarities, or unique interpretations.

In the final step, the lead researcher utilized a qualitative content analysis method that incorporated data reduction procedures to organize and confirm main issues that emerged [28]. A problem was deemed important if two or more participants identified an issue with a question or response category; however, individual issues were also noted and discussed. A written summary was produced to report on main issues and revisions that were required using similar categories reported in the literature: general design, culture, gender, and translation issues [27].

## Results

### Cross- cultural translation and adaptation

After two forward translations (T1 and T2) were completed, both translators met to review each translation. A small number of discrepancies were detected that related to correct translation and meaning during the synthesis meeting of T1 and T2 translations. For example, the word “recent” was translated differently in T1 and T2 translations. A decision was made to keep T1 translation of “more fresh” because it was more conceptually aligned to the English term.

During expert committee review, a bilingual clinical expert reviewed the synthesis document of T1 and T2 translations and compared it to the source English language survey. This allowed issues of translation to be further assessed, and the subsequent discussion of key translated terms that needed to be conceptually aligned with the source language. One issue arose during expert committee review from the synthesis report with the question: “Before this test was described, had you ever heard of a home stool test” as it was translated without the word “before”, and thus the meaning differed. Therefore, the wording was changed to be clear on what the question was asking with the inclusion of a term that made the question conceptually aligned.

Another issue was the use of different translation terms for a specific question. The translated word for “not as bad” was “difficult” and assessed to be too harsh; therefore, it was decided to literally translate the word “bad” rather than keep the translated term “difficult”. Another example of an issue that arose during expert committee review was the omission of key terms, such as “not sure” in applicable response categories. Consequently, this term was translated and inserted into the appropriate places in the survey. Incorrect translation of “yours” was also identified, as it was translated as “mine”; this was corrected in the survey.

Another translated term discussed was “painful” as it was translated to be “tortuous”, which was considered to be too severe, and it did not truly represent the context of screening colonoscopy. Therefore, the expert committee review discussion led to the decision to replace the translated term for “painful” to a term that translated into “less severe”. A final issue that emerged was the use of “professional” in the context of health professionals. Discussion led to the resolution that the term was representative of medical professionals not professionals in general, and thus was retained. While the expert committee review revealed key issues, some translated terms would need to be tested during cognitive interviews to assess conceptual understanding. For example, the literal translation of “bad” would have to be assessed to determine if it was correctly understood as intended.

### Cognitive interviews

A total of 30 participants completed an interview that was on average an hour in duration. Age, gender and years in Canada were similar across Urdu and English language participants. Mean age of participants in both groups was 59 years. Over 50% of participants’ self-identified country of origin as Pakistan with the remaining from India. All participants had a family physician, and of these, 63% reported their physician was of the same cultural background. See Table 2 for socio-demographic characteristics.

**Table 2:** Participant Socio-demographic Characteristics

Variable	Language Group	
	Urdu (*n* = 18)	English (*n* = 12)
Age: Mean, Standard Deviation	58.8(7.31)	59(6.2)
Sex, % (n)		
Male	44.5(8)	58.3(7)
Female	55.5(10)	41.6(5)
Country of birth, % (n)		
India	33.3(6)	58.3(7)
Pakistan	61.1(11)	41.6(5)
Bangladesh	0(0)	0(0)
Other	5.5(1)	0(0)
Years in Canada, % (n)		
More than 30 years	16.6(3)	25(3)
21–30 years	11.1(2)	8.3(1)
11–20 years	61.1(11)	50(6)
6–10 years	0(0)	16.6(2)
Less than 1 year	11.1(2)	0(0)
Highest completed education, % (n)		
Less than high school	22.2(4)	8.3(1)
Completed high school	22.2(4)	8.3(1)
Completed some college/university	22.2(4)	0(0)
University degree	16.6(3)	33.3(4)
Post graduate degree	16.6(3)	50(6)
Had a family physician, % (n)	100.0(18)	100.0(12)
Family physician from same cultural, % (n)		
Yes	55.5(10)	75(9)
No	27.7(5)	16.6(2)
Not applicable	16.6(3)	8.3(1)

Scripted and emergent *verbal probing* elicited comments and feedback among Urdu and English language speaking SA immigrants. While the use of *think-aloud* technique was avoided due to prior reports of difficulties with this technique among some ethno-cultural populations [11, 29], a good proportion of participants’ provided *think-aloud* comments on their own in all cognitive interview rounds. Based on Willis [21], the use of *verbal probes* may come to bear a resemblance to *think-aloud* techniques. Some participants’ spontaneously responded with their thoughts about the rationale for responses or opinions. Consequently, *think-aloud* responses were recorded for items that did not have scripted *verbal probes* during the interviews. Approximately 56% of these items had comments in Urdu language surveys and 63% of items for English language surveys.

### Interpretation of qualitative data

The interview summaries included qualitative data (e.g. notes logged by interviewers) and provided the main data source for analysis. The analysis focused on item level results of each individual interview, and aggregated results across each round of interviews. Qualitative data of the practice round and round one were combined during analysis because no problems aside from word or format issues were encountered in the practice round. Using a *joint collaborative analysis* process, interviews were analyzed separately for Urdu and English language results. After round one revision, we conducted round two. As we were hearing similar beliefs and ideas as in round one and no new major problems emerged in round two, we did not believe it was necessary to do another round of interviews [24]. In the following discussion, the interpretive analysis of data will: (1) discuss findings related to the 12 scripted *verbal probes*; (2) report on common or unique themes reflecting beliefs or ideas that emerged from respondents; and (3) present key issues that were identified and compared across both the Urdu and English language surveys.

### Findings from scripted *Verbal Probes*

The 12 scripted *verbal probes* testing comprehension and recall of selected items were incorporated within the cognitive interview protocol to address key issues during survey development. An expert advisory group from public health and an expert measurement committee provided input on the probes utilized in this study [3]. The following highlights common and unique responses across cognitive interviews for all rounds.

#### Introductory statement: Home stool test

The description of the home stool test, including “fecal matter or stool” concepts where assessed to test comprehension and interpretation. Among Urdu and English language groups in all rounds, there was a good understanding that the test was for examining “bodily waste” and checking if stool was “normal” or “abnormal”. The rationale for testing was also clear in that it was to “check for blood”, and was required to find cancer at an “early stage so it can be cured”. Only one female English language participant felt “awkward” about talking about feces as it was a sensitive topic.

#### Item: Heard of test and testing of response category

For participants who responded with “Not sure/do not know” in all rounds, they were probed on the meaning of these concepts. Overall, there was good comprehension in what this response category was asking.

#### Item: Most recent home stool test

There was good comprehension of what the item was asking participants as responses indicated that it was “clear”, and “not difficult”. Common responses to probing across all rounds resulted in responses that the confidence and estimation of the time when one had the home stool test was “not hard” “easy” and “not difficult” to recall. Male and Female English and Urdu language participants felt “confident” or were “100%” sure of their response.

#### Item: Most recent colonoscopy

Similar responses to confidence and estimation of a colonoscopy test were identified across both language groups in all rounds. In the English language group, female and male participants felt “confident” in the estimation of dates and believed it was an “easy” or “not difficult” question. Only one female English language participant commented that she could remember the year but not the exact date of her colonoscopy.

#### Scale item: I feel I will get colon cancer in the future

Participants’ in both language groups across all rounds commented on their understanding of this question as a “chance” of getting colon cancer. The meaning of the concept “feel” elicited responses such as “belief”, “sense”, “think”, “perceive”, and “possibility”. One female Urdu language participant believed that if she went for screening, she may not get colon cancer, while another male Urdu language participant felt that it was a serious disease. Having a family history or symptoms was also mentioned by male and female Urdu language participants as risk factors for colon cancer. One male Urdu language participant did not believe he was at risk for colon cancer because of his culture and family history. Another male English language participant responded with a comment that because he was “trying to eat healthy”, he did not believe he would get colon cancer.

#### Scale item: I am more likely than the average person to get colon cancer

The beliefs and ideas participants provided in their responses across all rounds consistently reflected comprehension and understanding of the item. Participants believed that this disease could affect “normal people” in “good health” or “healthy”, and of the same “age” or “gender”, and those “50 plus”. A male Urdu language participant believed that because he did not have a family history, he did not have a “chance” of getting colon cancer. One male English language participant stated that his family gave him “healthy foods”, a preventive measure. Another male English language participant indicated that because of his religious beliefs (Muslim), his hygiene practice of washing after a bowel movement was a health practice protecting against colon cancer.

#### Scale item: When I think about colon cancer, my heart beats faster

Across all rounds, participant responses to this item and terms “heart beats faster” reflected the emotional response that colon cancer may perpetuate, feelings such as “fear”, “panic”, “nervous”, “terrified”, “upset”, and “shock”. A female Urdu language participant believed that she was “not scared” because colon cancer could be cured, while a male English language participant believed that people should “consider” having the test.

#### Scale item: Colon Cancer would threaten a relationship with my partner

There was great understanding of the meaning of this item and the comprehension of the concept of “threatens” among all participants. Common beliefs were that the impact of a colon cancer diagnosis would pose a risk to the “mental”, “physical”, and instrumental (i.e. finances) aspects of the relationship. Other terms used to describe this were “danger”, “harm”, “separation”, and “break-up”. Two Urdu language participants (male and female) reflected on the fact that they understood and believed that colon cancer was “not contagious”.

#### Scale item: If I had colon cancer, my whole life would change

Across all rounds, participants’ comprehended the meaning that if someone was diagnosed with colon cancer, life would be “different”, and that it would impact life on an emotional and psychological, physical, social, and instrumental level. This included feeling “no hope”, being “stressed”, suffering “depression”, and experiencing “pain”, other “health problems” or having to do more treatments. As well, the social aspect included being treated differently, or the medical care required would take a financial toll on one’s life. A female Urdu language participant did not believe life would be affected because of advances in science, the ability “to cure many major diseases”, and that it would “be easy to cure”. A female English language participant’s rationale for her response related to not having enough information about colon cancer, while another female English language participant’s response reflected her “religious beliefs”.

#### Scale item: The treatment for colon cancer may not be as bad if the cancer is found early

Comprehension of this item and key concepts reflected good understanding across all rounds of testing. Participants believed that colon cancer was “easy to cure” as long as it was found early. There was this belief that the treatment would not be as “hard”. As well, treatment would not require “extensive surgery”, recovery would be “easier”, and early treatment would be “successful”. The concepts, “not as bad”, were interpreted as “less painful”, “less upset”, or “not as hard” than if cancer was found at a later stage.

#### Scale item: The cost would keep you from having a home stool test

The common belief across all rounds in relation to “cost” was that it would involve paying for the home stool kit, and as a result, potentially not having the test. Both male and female Urdu language participants indicated that the item meant if one had to pay for the test, it would be avoided, or would not be done. Both English and Urdu language participants indicated that they knew that the test was covered by the publically funded healthcare plan. Several male English language participants considered “cost” as also the “time” to do the test, and taking “time off from work”.

#### Scale item: I am confident that testing three separate bowel movements would not be inconvenient

Common responses in relation to comprehension and understanding of testing and the concept “inconvenient” from all rounds reflected the belief that this would be “difficult”, “not easy”, “uncomfortable”, “too much trouble with 3 samples” and that there would not be “time”. Both language groups indicated that the inconvenience would also relate to one’s “busy schedule” including work demands or lack of time in the day. A female Urdu language participant recognized that her schedule was busy; however, she would still do the test because she wanted “to be healthy”.

### Common themes

#### Doctor recommendation or advice

An interesting observation that arose from the cognitive interviews included participants’ *think-aloud* responses in relation to their family doctor. In round one, there were equal mentions of the doctor among both Urdu and English language interviews (Urdu *n* = 14, English *n* = 15); however, in round two, English language interviews had 13 comments versus only four comments from Urdu language interviews. These findings highlight the importance of the family doctor in either seeking out information, advice, or playing a role in screening recommendations. Furthermore, prior research supports the importance of physician recommendation for access to cancer screening [1].

#### No symptoms

For a number of items, male and female English language participants provided rationale for their responses based on the belief that they did not have bowel problems or symptoms. There were 10 comments (round 1, *n* = 8; round 2, *n* = 2) reflecting this belief.

#### Risk factors

An equal number of English and Urdu language participants (*n* = 20, males and females) also rationalized responses by providing an indication that risk factors associated with colon cancer were well understood. These participants reported that they had healthy diets, walked or exercised, understood family history as a risk factor, and recognized the value of regular check-ups with their physicians.

### Key issues

#### General design issues

Qualitative analysis revealed a number of general design concerns equally for Urdu and English language interviews (Table 3). One issue was that several participants had not initially realized that the term, “home stool test” related to screening; however, they were aware of having the test ordered by their physician at some point in the past. Therefore, we added interviewer instructions in the survey for the knowledge item in the survey related to having heard about CRC screening. Another issue related to the position of an item in the survey when participants had limited awareness of other colon cancer screening tests, such as colonoscopy; the item was relocated to a position after the introduction of the colonoscopy test.

**Table 3:** General Design Issues and Revisions

General Design		
Issue & Survey	Description	Revisions
Skip pattern	*Knowledge Item:* “Before this test was described, had you ever heard of a home stool test?” Two participants, one in each group indicated that while they had not heard of the home stool test and perhaps did not recognize it, they had the procedure at some time. This was uncovered later in the survey after they had already responded to the first item.	Cognitive interviewer instructions were inserted after this item: “If participant states NOT HEARD but states they HAD the test, go to question that asks if they had the test and revise.”
*Urdu language*		
*English language*		
Format and position of item in survey	*Colon Cancer Practice Item*: “Do you plan to go for any other type of colon cancer screening test in the future?” was problematic for a number of participants (Urdu, *n* = 3) because at this point in the survey, colonoscopy had not been explained. These participants provided comments that they did not know about any other tests. Additionally, during *joint collaborative analysis,* it was determined that participants provided the rationale that they would only do a test with physician recommendation. Given that the test requires a doctor’s order in Canada, it was noted that additional information regarding this would be important to include.	This item was relocated after the description of colonoscopy and related items. Modification of the item was also indicated to include doctor advice. “Do you plan to go for any other type of colon cancer screening test if your doctor ordered the test (for example, a colonoscopy)?”
*Urdu language*		
Missing response category	*Socio-demographic Item:* “In your household, tell us the individuals who make up your family: Mark all that apply.” Most participants in both groups stated they also lived with children. This was not included in the response categories for the socio-demographic item on household. During the interview, the cognitive interviewer added this with written notes.	An additional response category was added: “Living with own children”.
*Urdu language*		
*English language*		
Response category	*3 Scale Items (Percieved barriers)*: “Having a colonoscopy is painful.” “Having to follow a special diet and taking a laxative would keep you from having a colonoscopy.” “You are afraid to have colonoscopy because of the possibility there may be bleeding or tearing of the colon.” English language participants who lacked knowledge provided additional comments that they “did not know”. Responses were in line with what was expected in these items, which was “I neither agree or disagree”.	No revisions were indicated. The rationale was that participant’s responses were in line with the goals of testing the items. Only English language participants and no Urdu language participants had this issue related to their not having had a colonoscopy.
*English language*		
Item relevance	*2 Scale Item (Perceived barriers): *“The cost would keep you from having a home stool test.” “The cost would keep you from having a colonoscopy.” The cost were clear for all participants. One issue arose for both Urdu and English language participants; their general knowledge of the test was that it was paid for by the Ontario Health Insurance Plan (Round 1 English, *n* = 3; Round 2 Urdu, *n* = 3 and English, *n* = 2). During *joint collaborative analysis* discussions, it was felt that some participants’ may be confused and not know if there was a cost, especially if they lacked knowledge.	No revisions indicated. Further discussion was undertaken and it was decided to retain these items so that further testing could be done through pilot testing with a larger population.
*Urdu language*		
*English language*		

Another design issue was related to the exclusion of children in the response categories of an item concerning the number of family members in the household. Given the collectivist nature of SA immigrants, it was important to add another response category to include the possibility that participants lived with their children. Revisions were made to address these issues in the survey and no further issues arose in round two (Table 3).

#### Cultural issues

Several issues emerged with items related to different beliefs across English and Urdu language groups (Table 4). One issue arose with a *subjective norm* item regarding the influence of family and friends on CRC screening decision-making. A number of participants believed that their doctor had greater influence and that they would not discuss CRC screening with family or friends, and responses reflected these perceptions. The suggestion was made by one participant to add a “not applicable” response. No modification was made because recommendations have postulated that “not applicable” responses compromise data quality and analysis, as well having this option tends to increase its selection in surveys [30]. Most importantly, participants would not employ cognitive processes to come to a response and this in turn may lead to satisficing results.

**Table 4:** Cultural Issues and Revisions

Cultural Issues		
Issue	Description	Revisions
Culturally appropriateness of item wording	*Scale Item (Seriousness):* “Problems I would experience with colon cancer would last a long time”. One participant who completed the English language version indicated that the wording was suggestive and intoned that the individual had colon cancer, which was a culturally sensitive issue for those who believed there were connotations to being diagnosed. As well, quite a number of Urdu language participants (*n* = 5) also used “if” in their qualitative statements.	To better place this item in the context of the meaning, and make it more culturally appropriate, the wording was modified to: “Problems I would experience *if I* had colon cancer would last a long time”. This was tested in round 2, and no further problems emerged.
*Urdu language*		
*English language*		
Topic not discussed among family members	*Scale Item (Subjective norm):* “Members of my immediate family think I should have colon cancer screening.” Among English language participants (*n* = 3), comments indicated that the topic of colon cancer was not discussed in the family. However, for two participants, the responses were in line with their selection. Only one participant suggested having a “Not applicable” option.	No modifications were made to the response categories as doing so would compromise data quality and analysis.
*English language*		

The item “Problems I would experience with colon cancer would last a long time” was found to be culturally inappropriate for participants, because it inferred a diagnosis of colon cancer. Therefore, the item was modified to be more culturally sensitive and reflect the possibility of a cancer diagnosis. No major issues with this item arose during testing in round two.

#### Gender issue

An issue arose with both the Urdu and English language interviews, whereby, male participants’ responded to gender preferences for a health care provider, an item that was intended for female participants’ (Table 5). Prior studies with SA immigrant women reported on the preference for health care providers of the same gender [1]; therefore, we decided to include this item for response by females only. In *joint collaborative analysis*, it was discovered that cognitive interviewers were not adhering to the prompt “Female only” for the item. Placement of the item towards the end of the survey may have been a factor in this oversight. The item was revised to be inclusive of both male and female preferences for gender of health care provider. Minor re-wording modification addressed this issue for round two.

**Table 5:** Gender Issue and Revision

Gender Specific Issue		
Issue & Survey	Description	Revisions
Item wording was gender specific	*Socio-demographic Item:* “If you are female and have a male physician, would you prefer a female health care provider, such as a female doctor or female nurse practitioner for health exams?” Responses categories included male, female, and do not care. While this item was intended for only females to respond, a number of males (Urdu, *n* = 2; English, *n* = 2) responded to the item indicating that they preferred a male. Both cognitive interviewers inadvertently asked this item because we would not have picked up on this issue.	The item was reworded for round two to include both male and female participant responses: “For health exams, would you prefer a health care provider who is?” The responses categories also were modified to: male, female, and no preference.
*Urdu language*		
*English language*		

#### Translation issue

In the Urdu language surveys, no translation issues were uncovered during any of the rounds. During *joint collaborative analysis*, only one translation issue in the English language survey was revealed with the term “inconvenient” (Table 6). *Verbal probes* involved participants frequently paraphrasing the term “inconvenient”; however, there were times when participants repeated the word with no elaboration of the meaning because they were not probed to consider another word to describe the term. During *joint collaborative analysis* discussion, it was decided that rather than change the word “inconvenient”, interviewer instructions for other terms used by participants would be added to the protocol. These instructions would guide the cognitive interviewer to probe the participant further to elicit more meaningful responses. In round two, the addition of these instructions resolved the issue because the responses resembled paraphrasing of the term similar to Urdu language participant responses in round one.

**Table 6:** Translation Issue and Revision

Translation		
Issue & Survey	Description	Revisions
English source language term did not incite further elaboration in probing as did the translated term	*Scale Item (Self-efficacy):* “I am confident that testing three separate bowel movements would not be inconvenient.” The word “inconvenient” was translated as “hard to do” in the Urdu language version during translation. “Inconvenient” is an English term to convey problematic or difficulty with a task. This was comprehensible in Urdu and resulted in participants using other meaningful responses during *verbal probes* such as: “difficult for me”, “not easy to take test on different time because I am very busy and have day care”, “a problem”, “not easy, takes time”, and “takes me out of my way to get it”. In English, the word “inconvenient” did not provide as meaningful responses to verbal probing, for example: “too much trouble”, and “uncomfortable” were terms used. Upon further discussion at the *joint collaborative analysis* meeting, the cognitive interviewer indicated that “uncomfortable” related to the stool collection process and the impact on the individual to do this in a given day.	No modification was made to the English version of the item. As this was a minor issue, it was decided that providing additional training to interviewers on non-leading probing was satisfactory. Therefore, interviewer instructions were added to stimulate more meaningful responses, particularly for English language participants. We used Urdu language participants’ responses to refer to in terms of the appropriate probing descriptors that should emerge.
*English language*		

## Discussion

The development of the Colon Cancer Screening Behaviours Survey involved a multi-stage process that valued the perspectives of the SA population, was informed by findings from prior studies [1, 2], and incorporated pre-existing measures that required additions and modifications [3]. This study adds to the literature by cognitively pre-testing an Urdu and English language version of the Colon Cancer Screening Behaviours Survey for future field-testing, research, and use among SA populations.

Cross-cultural translation and adaptation of measures into another language have been undertaken in a number of studies [31–33]; however, no prior studies have been found that cross-culturally translated and adapted a colon cancer survey into the Urdu language. In one study [34], a CRC screening questionnaire was translated into Hindi and Gujarati using a bilingual-bicultural committee translation approach derived from translation methods proposed by Harkness and Mohler [35]. Likewise, in another study [36], a CRC screening survey assessed behaviours among English speaking SA’s in New York/New Jersey, but no information was provided on whether it was culturally adapted for this SA population. The use of cross-cultural cognitive interviewing [10] in this study enhanced rigor, and provided details to guide future scholarly work.

In our study, cross-cultural equivalence was assessed by determining if interpretations varied by two distinct language groups (English and Urdu). Literature reporting on pre-testing of colon cancer screening measures that used standard cognitive interviews among diverse samples are available [37, 38], although, comprehensive details of the processes are not always provided [39]. In studies that have conducted cognitive interviews for CRC screening measures, the populations included veterans, African American, Native American, Asian and White samples from the USA, consisting of 18–36 participants, and methods used retrospective probing [37, 38]. Retrospective probing has limitations because participants may not recall what they were thinking when they responded to a specific item earlier when the survey question was initially administered and this could lead to fabricating a probe response [21]. Cross-cultural cognitive interviewing has been undertaken to explore other health related topics (i.e. work life, dietary, general health survey) among different ethno-cultural groups and provided substantial details of the process [20, 26, 40].

Scripted *verbal probes* were effective in the cognitive interviews with Urdu and English language speaking SA immigrants. Participants were enthusiastic and responded to *verbal probes* that provoked follow up to their responses. Participants provided rationale for their responses, or comments that took on aspects of *think-aloud* techniques [16]. This was a valuable finding for those conducting cross-cultural cognitive testing of survey measures with SA populations.

The nature of general design issues were simple to correct and were equally evident in both the English and Urdu language surveys. Culture specific issues included culturally sensitive wording. English language speaking participants in round one who were long term residents of Canada, preferred not to discuss colon cancer and screening with their family compared to Urdu language speaking participants. This revealed the diversity of individual beliefs and practices in the cognitive interview sample. Furthermore, this finding aligns with prior studies, where some SA immigrants did not believe it was appropriate to discuss cancer or screening with family or friends [1], while others believed that family was supportive in health decisions including CRC screening [1, 2]. This highlights the need to recognize diversity and assess individual preferences among members of diverse ethno-cultural groups. The *subjective norm* measure aims to distinguish the influence of family in decisions around colon cancer screening.

A gender specific issue also arose during round one regarding gender preferences of one’s family physician. Prior research uncovered preferences for a female physician among female SA immigrants [1, 2]. While it was not the intent of the developers to exclude the male perspective, it was beneficial that cognitive interviews uncovered this issue. Eliciting information about SA male participant’s preference is important in that it may affect health seeking and colon cancer screening behaviours, and presents an opportunity for further exploration through research.

The limited translation issues that arose during cognitive interviews may have resulted from the evidence-based cross-cultural translation and adaptation approach used in this study. Any issues that arose during the three stage process of cross-cultural translation and adaptation were dealt with at each stage through revisions.

The cognitive interviews were conducted face-to-face in the field. Participants were interviewed in their home or community, which is preferable to a cognitive lab because it emulates real survey administration practices that are likely to take place when the survey is pilot tested [23]. Cognitive interviews that deal with sensitive topics are best conducted in the field to facilitate more comfort, accuracy, and encourage more truthful responses [21]. The cognitive interview process proved to be successful among SA immigrants who completed the Urdu and English language version of the survey. This supports findings from one review that most studies employing scripted *verbal* and emergent *probing* were effective across diverse language and cultural groups [10].

The use of a *joint collaborative analytic* process was considered to be a favourable method in comparing results within a group and the decision-making process involved in assessing the function of each item individually for each interview [10]. This strategy was beneficial due to the limited experience of interviewers as it created a feedback loop and continued communication. During interviews, the lead researcher was in constant communication with interviewers to provide support, to share information, and to assess if there were any issues. There were numerous meetings held for training and *joint collaborative analysis* (*n* = 6). The goal was to work together for the purpose of further processing of data, interpretation, and aggregation [27]. Depending on the experience of interviewers, the continuing support by the lead researcher may prove more beneficial in detection of issues for those conducting research with novice cognitive interviewers [10].

### Limitations

Our decision to stop at 30 cognitive interviews may be seen as a limitation, however, we believe that the final survey is improved because of the rigorous cross-cultural translation and adaptation and cognitive interviews because major problems were addressed. It is now ready for pilot testing with a larger sample so that measures contained within the survey may be psychometrically tested.

Notes were taken during and after the interview with no audio or video recording, which are additional methods recommended for cognitive testing [16]. The audio or video recording may have uncovered more problematic issues than handwritten notes alone. However, seeking permission to audio or video tape the interviews was not culturally appropriate because interviews were held in participant’s homes and communities.

Another limitation was the interviewers’ lack of experience with cognitive interviewing despite their fluency in both languages. However, this approach is acceptable in keeping with Willis’ [10] recommendations as long as the interviewer has access to scripted *verbal probes* and receives adequate training.

A fourth limitation was related to participant recruitment for cognitive interviews as there were 13% of participants who had been in Canada for less than 10 years. While an attempt was made to recruit participants who were recent immigrants, there were challenges due to lack of familiarity with the topic and comfort with the interviewer. A greater proportion of newcomer immigrants may have uncovered problems with the Urdu language survey as new immigrants may not be as familiar with preventive health practices such as cancer screening [41]. This may relate to socio-cultural adaptation in the new society. Those settled for less time may be in the process of continuing to develop socio-cultural knowledge and skills related to the adoption of preventive health practices [42]. A lack of familiarity with the term “screening” may have also been related to level of acculturation [37].

## Conclusions

In this study, we cross culturally translated and adapted the Colon Cancer Screening Behaviours Survey, and pre-tested it among Urdu and English speaking SA immigrants in Ontario, Canada. The use of rigorous methods for both cross-cultural translation and adaptation and cognitive testing were successful in assessing the conceptual basis of previously developed measures that were adapted, and modified among this diverse population. Revisions to the survey improved it, and now it is ready for field testing and for assessment of psychometric properties of key measures among the SA population.

## Additional file

Additional file 1: Appendix A Scripted verbal probes. (DOCX 25 kb)
