# Supplementary material for: Cognitive testing of the Colon Cancer Screening Behaviours Survey with South Asian immigrants in Canada
Source: J Patient Rep Outcomes. 2017 Oct 19;1:7. doi: 10.1186/s41687-017-0007-4 (PMC6091743; doi:10.1186/s41687-017-0007-4)
Supplement: Supplementary file 1 — Appendix A Scripted verbal probes. (DOCX 25 kb) [file 41687_2017_7_MOESM1_ESM.docx]

**Appendix A: Scripted Verbal Probes**

**1. Introductory statement**

I would first like to begin by asking you about screening for colon cancer.

Screening for colon cancer means having a test to check for cancer even if you do not have symptoms or problems with you bowels.

*The following questions are about the home stool test, a test to check for colon cancer. In your home, you take a sample of fecal matter or stool (poop) and follow the instructions to complete the test. The test checks for small amounts of blood that you cannot see in the stool.*

**PROBE (for statement above)**

**Can you tell me in your own words what this introduction is saying?**

**What, to you, does “fecal matter or stool” mean to you?**

**Results:**

**Suggested revisions:**

**2. Question:** “Before this test was described, had you ever heard of a home stool test?

**PROBE**

**Can you tell me in your own words what that question is asking?**

**ONLY ask people the question below who respond with “Not sure/do not know”**

**Tell me why you chose “Not sure/do not know”?**

**Results:**

**Suggested revisions:**

**3.** When did you do your most recent home stool test?

🞎 A year ago or less

🞎 More than 1 but not more than 2 years ago

🞎 More than 2 but not more than 5 years ago

🞎 More than 5 years ago

🞎 Not sure/ do not know

**PROBE**

**Can you tell me in your own words what the question is asking?**

**How sure of you of your answer?**

**How hard was this to answer?**

**Results:**

**Suggested revisions:**

**4.** Was your most recent colonoscopy:

- A year ago or less
- More than 1 but not more than 2 years ago
- More than 2 but not more than 5 years ago
- More than 5 years ago
- Not sure/ do not know

**PROBE**

**How sure of you of your answer?**

**How hard was this to answer?**

**Results:**

**Suggested revisions:**

**5. Scale item: I feel I will get colon cancer in the future.**

| **PROBE**  **Can you repeat the question in your own words?**  **What, to you, does “feel” mean?**  Alternate way to pose question: What did you understand by the word “feel”?  **Results:**  **Suggested revisions:** |
| --- |

**6. Scale item:** I am more likely than the average person to get colon cancer.

| **PROBE**  **Can you repeat the question in your own words?**  **What, to you, does “average person” mean?**  **Results:**  **Suggested revisions:** |
| --- |

**7. Scale Item: When I think about colon cancer, my heart beats faster.**

| **PROBE**  **Can you repeat the question in your own words?**  **What, to you, does “heart beats faster” mean?**  **Results:**  **Suggested revisions:** |
| --- |

**8.** Colon cancer would threaten a relationship with my partner.

| **PROBE**  **Can you repeat the question in your own words?**  **What, to you, does “threatens” mean?**  **Results:**  **Suggested revisions:** |
| --- |

**9.** If I had colon cancer, my whole life would change.

| **PROBE**  **In your own words, what do you think this question is trying to ask?**  **What, to you, does “whole life would change” mean?**  **Results:**  **Suggested revisions:** |
| --- |

**10**. The treatment for colon cancer may not be as bad if the cancer is found early.

| **PROBE**  **Can you repeat the question in your own words?**  **What, to you, does “not as bad” mean?**  **Results:**  **Suggested revisions:** |
| --- |

**11.** The cost would keep you from having a home stool test.

| **PROBE**  **Can you repeat the question in your own words?**  **What, to you, does “cost” mean?**  **Results:**  **Suggested revisions:** |
| --- |

**12.** I am confident that testing three separate bowel movements would not be inconvenient.

| **PROBE**  **Can you repeat the question in your own words?** *(To test how well the participant comprehends the question)*  **What, to you, does “inconvenient” mean?**  **Results:**  **Suggested revisions:** |
| --- |
